# Effects of Thickness of a Low-Temperature Buffer and Impurity Incorporation on the Characteristics of *Nitrogen*-polar GaN

**DOI:** 10.1186/s11671-016-1727-8

**Published:** 2016-11-18

**Authors:** Fann-Wei Yang, Yu-Yu Chen, Shih-Wei Feng, Qian Sun, Jung Han

**Affiliations:** 1 Department of Electronic Engineering, Southern Taiwan University of Science and Technology, Tainan, Taiwan, R.O.C.; 2 Department of Applied Physics, National University of Kaohsiung, No.700, Kaohsiung University Rd., Nan Tzu Dist., 81148 Kaohsiung, Taiwan, R.O.C.; 3 Key Laboratory of Nanodevices and Applications, Suzhou Institute of Nano-tech and Nano-bionics, Chinese Academy of Sciences, Suzhou, China; 4 Department of Electrical Engineering, Yale University, New Haven, CT USA

**Keywords:** *N*-polar GaN, Buffer layer, Thickness, Biaxial strain, Impurity incorporation

## Abstract

In this study, effects of the thickness of a low temperature (LT) buffer and impurity incorporation on the characteristics of *Nitrogen* (*N*)-polar GaN are investigated. By using either a nitridation or thermal annealing step before the deposition of a LT buffer, three *N*-polar GaN samples with different thicknesses of LT buffer and different impurity incorporations are prepared. It is found that the sample with the thinnest LT buffer and a nitridation step proves to be the best in terms of a fewer impurity incorporations, strong PL intensity, fast mobility, small biaxial strain, and smooth surface. As the temperature increases at ~10 K, the apparent donor-acceptor-pair band is responsible for the decreasing integral intensity of the band-to-band emission peak. In addition, the thermal annealing of the sapphire substrates may cause more impurity incorporation around the HT-GaN/LT-GaN/sapphire interfacial regions, which in turn may result in a lower carrier mobility, larger biaxial strain, larger bandgap shift, and stronger yellow luminescence. By using a nitridation step, both a thinner LT buffer and less impurity incorporation are beneficial to obtaining a high quality *N*-polar GaN.

## Background

III-Nitride semiconductors have been used for light-emitting diodes (LEDs), laser diodes, solar cells, and high electron mobility transistors (HEMTs) [1–4]. These nitride devices are generally grown along the polar *c*-axis (*Ga*-polar), where the built-in polarization field decreases the overlap of the electron and hole wave functions and leads to quantum-confined Stark effect (QCSE) [1–4]. Meanwhile, the reverse polarization field of *Nitrogen* (*N*)-polar III-nitrides along the -*c*-axis [000-1] can be used for device applications, such as enhancement mode and highly scaled transistors, photodetectors, Zener tunnel diodes, and sensors [5–7]. Due to the reversed direction of the polarization field, a larger forward bias reduces QCSE in *N*-polar quantum wells (QWs), increasing overlap of electron and hole wave function [4]. By time-resolved electroluminescence measurement, an *N*-polar LED has been shown to have more efficient carrier relaxation and faster carrier recombination [4].

The properties of *Ga*- and *N*-polar GaN are different in the direction of the polarization field, the incorporation of residual impurities, and the surface morphology [8–11]. Because of the low-quality epitaxial layers of *N*-polar GaN, the device performance of the *N*-polar LED is poor compared to that of the *Ga*-polar LED [4]. The incorporation of oxygen in *N*-polar GaN during nitridation is much larger than that in *Ga*-polar GaN [10, 11]. Oxygen incorporates on nitrogen sites by bonding with neighboring Ga atoms and substitutes for nitrogen atoms on the *N*-polar GaN surface [10]. By calculating the adsorption energy of oxygen on the different surfaces, atoms impinging on a group V site form three bonds to the *Ga* surface atoms, leading to a stronger bonding of oxygen atoms to the *N*-polar surface [11, 12].

In addition, hexagonal hillocks on a rough surface are often observed in *N*-polar GaN [13–15]. Smooth *N*-polar GaN grown on on-axis *c*-plane sapphire substrates can be achieved by the following approaches before the main GaN growth: (1) With a nucleation layer deposited at 550 °C, followed by an annealing at 1030 °C [16]; (2) With a nitridation step on the sapphire substrates [17]; and (3) By using a low temperature (LT) GaN buffer and optimizing the nitridation temperature of the sapphire substrates from 1130 to 950 °C, atomically smooth *N*-polar GaN has been achieved and the hillock density has decreased [6]. The GaN nucleation layers were deposited at 600 °C, followed by the main GaN layer at 1055 °C. Furthermore, an improved surface morphology and narrow spectral width can be obtained by growing *N*-polar GaN on misoriented sapphire, silicon carbide, and silicon substrates [18]. An optimized LT GaN buffer is crucial to improve the epitaxial layer quality of the multiple quantum well structures. However, the effects of the thickness of a LT buffer and impurity incorporation on the characteristics of *N*-polar GaN with either a nitridation or thermal annealing step were not well studied.

In this study, the effects of the thickness of a LT buffer layer and impurity incorporation on the characteristics of *N*-polar GaN are investigated by atomic force microscopy (AFM), secondary ion mass spectrometry (SIMS), photoluminescence (PL), Raman, and Hall measurements. It is found that the sample with the thinnest LT buffer and a nitridation step proves to be the best in terms of a strong PL intensity, fast mobility, small biaxial strain, and smooth surface. Thermal annealing of the sapphire substrates may cause more impurity incorporation around the HT-GaN/LT-GaN/sapphire interfacial regions, which in turn may result in a lower carrier mobility, larger biaxial strain, larger bandgap shift, and stronger yellow luminescence. By using a nitridation step, both a thinner LT buffer and less impurity incorporation are beneficial to obtaining a high quality *N*-polar GaN.

## Methods

The *N*-polar GaN samples are grown on nominally on-axis *c*-plane sapphire in a metalorganic chemical vapor deposition reactor (MOCVD). Trimethylgallium (TMGa) and ammonia (NH_3_) are used as the precursors for Ga and N, respectively. Sapphire was heated up in a mixture of NH_3_ (3 slm) and N_2_ (4 slm) to a high temperature for nitridation. One *N*-polar GaN sample with 20-nm thickness of LT buffer (namely LT-20 sample) was grown on nitridized sapphire at 600 °C and a pressure of 300 mbar in H_2_, followed by the growth of ~1 μm *N*-face GaN at 1055 °C and 100 mbar with 0.5 slm NH_3_ and a TMGa flow of 66 μmol/min. Besides, the other two *N*-polar GaN samples with 21 and 32 nm thicknesses of LT buffers are prepared (namely LT-21 and LT-32 samples, respectively). The LT-21 and LT-32 sample sapphires did not have a nitridation step but had a thermal cleaning at 1070 °C in H_2_ for 2 min before the growth of LT-GaN buffer. The thermal cleaning of the sapphire substrates probably caused some issues at the initial growth interface, which accounts for the different properties among the LT-20, LT-21, and LT-32 samples.

The surface morphology was revealed by AFM (Park Systems, XE-70) with a non-contact mode using a silicon tip of curvature less than 10 nm. The samples were placed in a cryostat for temperature-dependent PL measurement with the 325-nm line of a 55-mW He-Cd laser for excitation. Raman spectra were recorded in the backscattering configuration using a Jobin Yvon-Horiba micro-Raman system (model T64000) with a 532-nm laser. Mobility and sheet resistance are measured by Hall/van der Pauw measurement.

## Results and Discussion

### AFM Images and SIMS Profiles

**Fig. 1:**
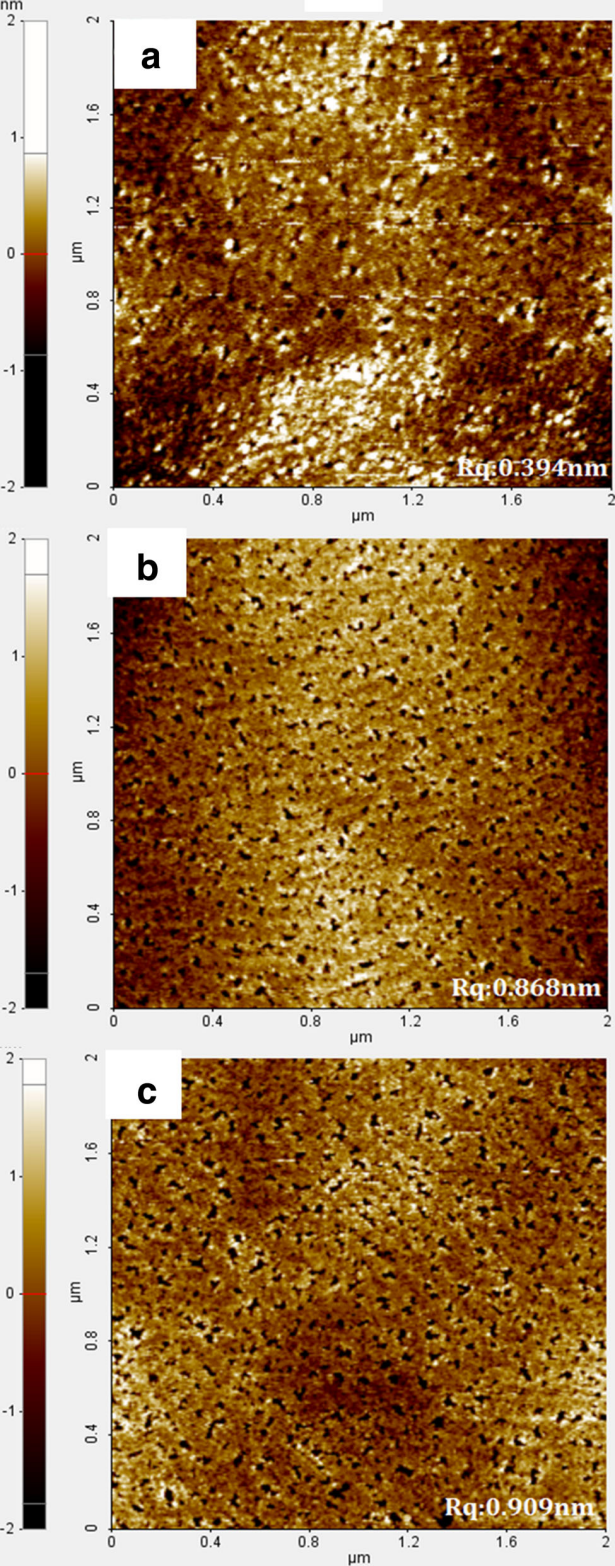
AFM images of the **a** LT-20 (Rq 0.394 nm), **b** LT-21 (Rq 0.868 nm), and **c** LT-32 (Rq 0.909 nm) samples. Surface roughness of each sample, Rq, is shown in the parentheses

Figure 1 shows the AFM images of the three *N*-polar GaN samples. The surface roughness for the LT-20, LT-21, and LT-32 samples are 0.394, 0.868, and 0.909 nm, respectively. For a thicker LT buffer, the surface morphology becomes rough. The smooth surface of the LT-20 sample represents the best sample.

**Fig. 2:**
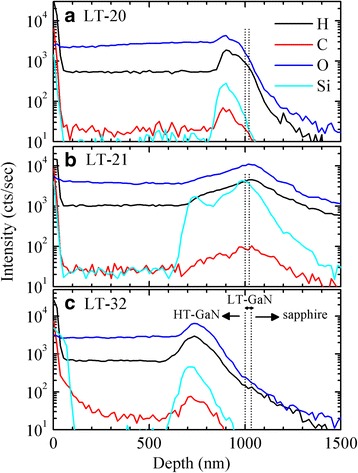
SIMS profiles of hydrogen, carbon, oxygen, and silicon incorporations for the **a** LT-20, **b** LT-21, and **c** LT-32 samples. The positions of HT-GaN/LT-GaN and LT-GaN/sapphire interfaces are indicated

Figure 2a–c shows SIMS profiles of the LT-20, LT-21, and LT-32 samples, respectively. The incorporations of hydrogen, oxygen, and silicon in the LT-21 and LT-32 samples without a nitridation step are higher than those in the LT-20 with a nitridation step. Especially, the impurity incorporations in the LT-21 sample are the highest and distributed more widely among the three samples. Without a nitridation step in the LT-21 sample, the thermal cleaning of the sapphire substrates probably may cause more impurity incorporation around the HT-GaN/LT-GaN/sapphire interfacial regions. Silicon probably comes from the susceptor SiC coating or reactor chamber ambient. Oxygen atoms can substitute for nitrogen atoms on the *N*-polar GaN surface [10]. The impurity incorporation affects the material and optical properties of *N*-polar GaN.

### PL and Raman Measurements

**Fig. 3:**
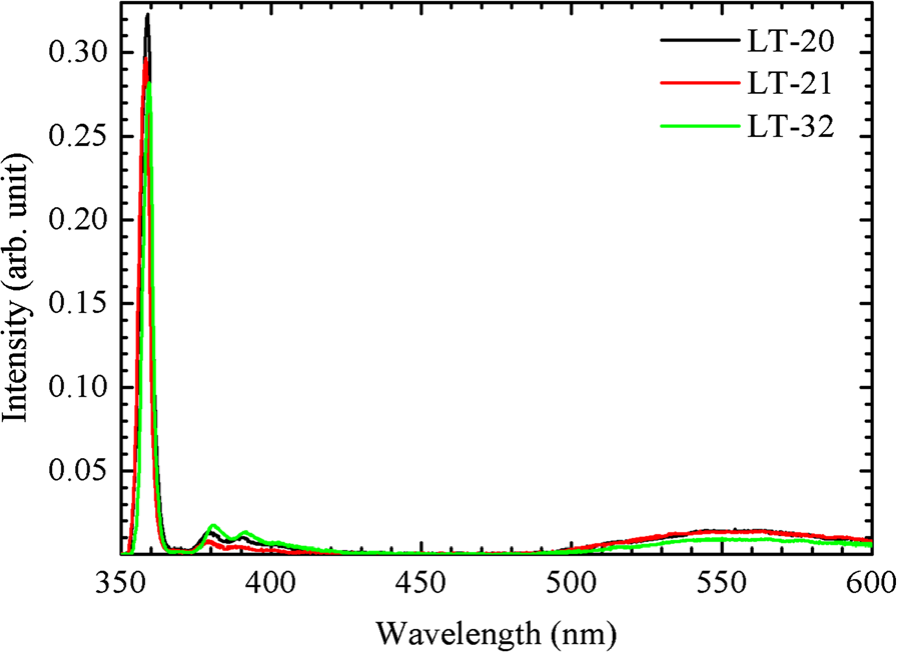
PL spectra at 10 K for the three samples

Figure 3 shows the PL spectra at 10 K of the three samples. The spectra show the band-to-band emission peak ~358 nm (3.464 eV), donor-acceptor-pair (DAP) band and LO phonon replica ~370–410 nm, and a weaker yellow luminescence for the three samples [11]. For a thicker LT buffer, the PL intensity is weaker. The DAP band and LO phonon replica for the LT-32 sample are stronger than those for the LT-20 sample, while those for the LT-21 sample are the weakest. The yellow luminescence for the LT-20 and LT-21 samples is nearly same, while that for the LT-32 sample is weaker. The substitution for nitrogen atoms by impurity atoms on the *N*-face surface creates more defect density. Hence, a higher impurity incorporation in the LT-21 sample leads to a stronger yellow luminescence.

**Fig. 4:**
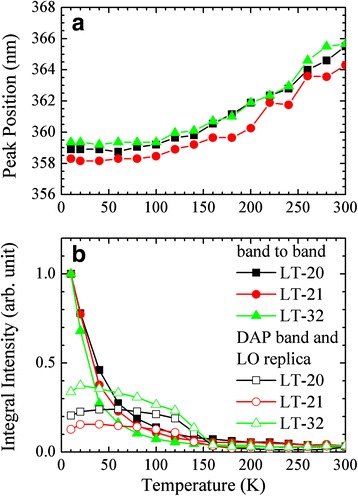
**a** PL peak position and **b** normalized integral PL intensity as a function of temperature for the three samples

Figure 4a, b shows PL peak position and normalized integral PL intensity as a function of temperature of the three samples, respectively. At higher temperature(s), the red-shift peak positions for all the samples are attributed to the band gap shrinkage [1]. Because of the large lattice mismatch between the films and the substrates, a large residual strain exists in the films. Strain relaxation is related to the thickness of the LT buffer. Hence, the bandgaps and PL peak positions of the three samples are different [1]. The strain inside the sample was determined by Raman measurement. In addition, as the temperature increases at ~10 K, the integral intensity of the band-to-band emission peak steeply decreases, while that of the DAP band and LO phonon replica becomes stronger, especially for the LT-32 sample. This shows that the apparent DAP band and LO phonon replica are responsible for the decreasing integral intensity of the band-to-band emission peak. The apparent DAP band and LO phonon replica are consistent with a poorer sample quality and rougher surface morphology in the LT-32 sample.

**Fig. 5:**
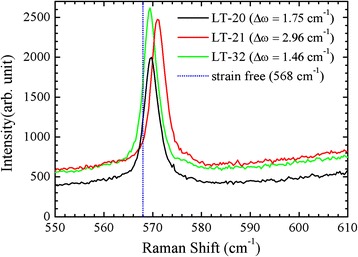
Raman spectra for the three samples at RT. The *dotted line* at 568 cm^−1^ show the strain-free *E*_2_-high mode for GaN [8]

Figure 5 shows Raman scattering spectra for the three samples. The spectra display *E*_2_-high mode of GaN. The dotted line (568.0 cm^−1^) shows the phonon frequency *ω*_0_ for the strain-free *E*_2_-high mode of GaN. Based on the deformation potential approximation, the phonon frequency shift, *Δω* = *ω* − *ω*_0_ = 6.2*σ*, can be approximated as a function of the biaxial strain, σ [19]. As shown in Table 1, except for the LT-21 sample, a thicker LT buffer helps strain relaxation, leading to a smaller residual strain. The largest biaxial strain, for the LT-21 sample, may be due to higher incorporations of hydrogen, oxygen, and silicon. It has been reported that oxygen incorporates on nitrogen sites by bonding with neighboring Ga atoms [10]. The larger oxygen atom distorts the atomic structure and results in a larger biaxial strain. In addition, with the relationship of the bandgap shift *ΔE*_*g*_(=0.0211*σ*) as a function of biaxial strain, σ [20], bandgaps of the three samples can be estimated. The energy bandgap of GaN is 3.39 eV at RT [21]. As shown in Table 1, the estimated bandgaps from phonon frequency shifts are nearly the same as the observed bandgaps from PL peak positions at 10 K for the three samples. It is suggested that more impurity incorporations inside the sample induce a larger biaxial strain and shift the PL peak position.

**Table 1:** Phonon frequency shift ∆*ω*, biaxial strain σ, bandgap shift ∆*E*_*g*_, estimated bandgap *E* ′ _*g*_ from phonon frequency shift, measured bandgap *E*_*g*_ from PL peak position for the LT-20, LT-21, and LT-32 samples

Sample	*∆ω*(cm^− 1^)	*σ*(GPa)	*∆E*_*g*_(eV)	*E* ′ _*g*_(eV) from Raman	*E*_*g*_(eV) from PL peak
LT-20	1.75	0.28	5.91×10^−3^	3.39581	3.3926
LT-21	2.96	0.48	1.012×10^−2^	3.40012	3.4037
LT-32	1.46	0.23	4.85×10^−3^	3.39485	3.3912

### Mobility and Sheet Resistance Measurements

Furthermore, Table 2 shows the PL intensity, mobility, and sheet resistance for the three samples. The characteristics of a strong PL intensity, fast mobility, and smooth surface of the LT-20 sample show the best sample. With more impurity incorporations inside the LT-21 sample, carrier-impurity atom scattering would slow down the mobility. In addition, the sheet resistance is inversely proportional to the film thickness [22]. Hence, sheet resistance in thicker GaN layer becomes lower.

**Table 2:** PL intensity, mobility (μ) calculated from Hall/van der Pauw measurements, and sheet resistance (*R*_sheet_) for the LT-20, LT-21, and LT-32 samples

Sample	PL intensity	μ (cm^2^/Vs)	*R*_sheet_ (ohm/□)
LT-20	Strong	98.100	3.069
LT-21	Medium	89.155	2.758
LT-32	Weak	92.11	1.592

## Conclusions

In summary, we have studied the effects of the thicknesses of a LT buffer and impurity incorporation on the material and optical properties of *N*-polar GaN samples. The sample with the thinnest LT buffer and a nitridation step proves to be the best in terms of a strong PL intensity, fast mobility, small biaxial strain, and smooth surface. As the temperature increases at ~10 K, the DAP band is responsible for the decreasing integral intensity of the band-to-band emission peak. In addition, the thermal annealing of the sapphire substrates may cause more impurity incorporation around the HT-GaN/LT-GaN/sapphire interfacial regions, which in turn may result in a lower carrier mobility, larger biaxial strain, larger bandgap shift, and stronger yellow luminescence. By using a nitridation step, both a thinner LT buffer and less impurity incorporation are beneficial to obtaining a high quality *N*-polar GaN.
